# A Framework to Automate Assessment of Upper-Limb Motor Function Impairment: A Feasibility Study

**DOI:** 10.3390/s150820097

**Published:** 2015-08-14

**Authors:** Paul Otten, Jonghyun Kim, Sang Hyuk Son

**Affiliations:** 1 Epic Systems Corporation, 1979 Milky Way, Verona, WI 53705, USA; E-Mail: pco890@gmail.com; 2 Department of Robotics Engineering, DGIST, 333 Techno jungang-daero, Hyeonpung-myeon, Dalseong-gun, Daegu 42988, Korea; 3 Department of Information and Communication Engineering, DGIST, 333 Techno jungang-daero, Hyeonpung-myeon, Dalseong-gun, Daegu 42988, Korea

**Keywords:** automated upper-limb assessment, Fugl-Meyer Assessment, low-cost sensors, machine learning, upper-limb motor impairment

## Abstract

Standard upper-limb motor function impairment assessments, such as the Fugl-Meyer Assessment (FMA), are a critical aspect of rehabilitation after neurological disorders. These assessments typically take a long time (about 30 min for the FMA) for a clinician to perform on a patient, which is a severe burden in a clinical environment. In this paper, we propose a framework for automating upper-limb motor assessments that uses low-cost sensors to collect movement data. The sensor data is then processed through a machine learning algorithm to determine a score for a patient’s upper-limb functionality. To demonstrate the feasibility of the proposed approach, we implemented a system based on the proposed framework that can automate most of the FMA. Our experiment shows that the system provides similar FMA scores to clinician scores, and reduces the time spent evaluating each patient by 82%. Moreover, the proposed framework can be used to implement customized tests or tests specified in other existing standard assessment methods.

## 1. Introduction

The prevalence of neurological disorders, such as stroke, cerebral palsy, and multiple sclerosis, has been rapidly increasing. For example, strokes affect up to 0.3% of the population every year in many countries [1], and is regarded as a typical target for motor recovery efforts because it substantially impacts patients’ quality of life.

Due to the large population of patients and the issue of medical costs, clinicians have a limited amount of intervention time that they can spend with each patient. Patient assessment is essential not only to quantify the severity of motor impairment, but to perform effective intervention as part of the process of recovery. Upper-limb motor impairment assessment is a time consuming process that must be done in person. For example, the Fugl-Meyer Assessment (FMA) [2], which is one of the most widely utilized clinical instruments for assessment, consists of 33 tests for the upper-limbs, where the clinician asks a patient to perform a series of pre-defined movements. It takes at least 30 min for a clinician to perform for each patient [2]. The Wolf Motor Function Test (WMFT) [3], Action Research Arm Test (ARAT) [4], and the NIH Stroke Scale (NIHSS) [5], and other assessment methods similar to the FMA also consist of many tests that are each rated according to the patient’s upper-limb motor functionality. Each of these tests also takes too much time to fully perform in clinical setting.

As a solution to the problem above, upper-limb motor impairment assessments need to be automated. Since automated assessments save time for clinicians, it also reduces medical costs by allowing clinicians to use their time more efficiently. In addition, this could make upper-limb assessment more frequent, resulting in better quality of patient care.

Several studies have been attempted to implement automated assessment systems. One study focused on the feasibility of automating FMA, but it required large and expensive setups, such as robotic arms, motion capture system, and EMG sensors [6], which are not suitable for a clinical setting. Another approach was to use accelerometers and a regression algorithm for the automated assessment [7]. Despite the low-cost setup and feasible assessment results, it could only automate a very small number of FMA tests (four out of 33 tests) due to the limited data acquired by the accelerometers [7]. The body-worn accelerometer has also been used to quantitatively index upper-limb motor function [8]. Recently, one study used a motion capture sensor, the Microsoft Kinect, with Principal Component Analysis (PCA) to automate assessment of some upper-limb movements which are part of FMA and ARAT [9], but still had much room for improvement for clinical use. The limitations of the Kinect sensor and PCA algorithm significantly reduced the number of FMA tests implemented. Moreover, the level of automation was not appropriate for a hospital setting, since a lot of the sensor data processing was not done in real-time.

This paper proposes a system framework that can be used to create a novel automated upper-limb assessment procedure as a time/cost saving measure for clinicians. The framework uses multiple low-cost sensors to reduce implementation cost and to collect enough sensor data to monitor various upper-limb movements. In order to score movements with the collected data (classification), we use machine learning algorithms because they work well with multi-sensor data for classification and are more extendable than the currently existing systems mentioned in terms of supporting new upper-limb movements. The framework also supports guidance materials to help patients perform the pre-defined upper-limb movements for the assessment without a clinician present, such as video instructions. This guidance is essential for improving the level of automation required for achieving the goal of a fully automated platform in a clinical setting. To demonstrate the feasibility of the proposed framework, we implemented a system based on the framework that can automate a large number of FMA tests. Through pilot experiments with eight healthy subjects and two stroke patients, we evaluated the accuracy and time efficiency of the system.

## 2. The Proposed Framework

**Figure 1:**
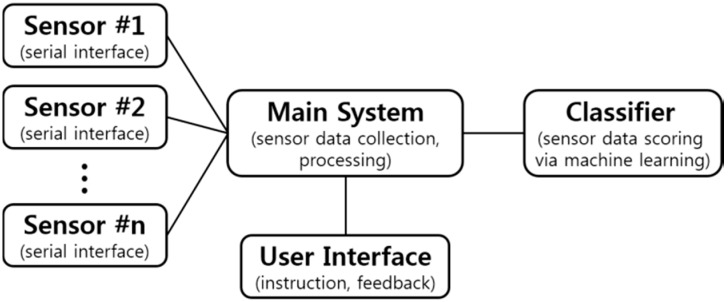
An illustration of the flow of execution for a system designed by the proposed framework.

**Figure 2:**
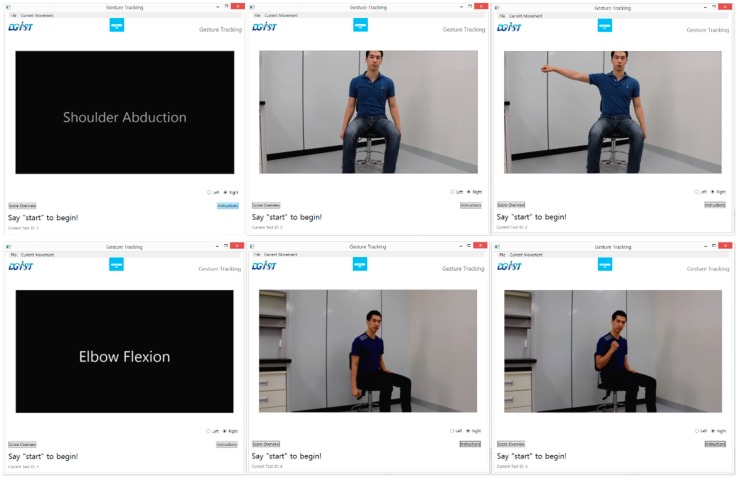
Example frames from an instruction video that is displayed to subjects. They are expected to follow along to perform each test movement.

The proposed framework is an outline that uses sensor data from low-cost sensors to collect data from patient movements. The sensor data is then processed and classified in order to generate a score of the patent movement for upper-limb assessment. An illustration of this design is shown in Figure 1. Typically, this framework will be used to develop systems deployed in a relatively controlled setting in a hospital. The possible scenario is as follows: The patient sits in a chair with sensors (*i.e.*, a motion sensor positioned in front of the patient who wears a glove sensor) and a user interface. The user interface instructs the patient to perform a test movement (likely by displaying a video as shown in Figure 2). While performing the movement, the system collects sensor data and scores it. The use of this system would result in more frequent and cost-effective patient assessments.

The scoring function of the proposed framework can be broken down into four main parts: Sensor data collection, sensor data pre-processing, feature extraction from the sensor data, and machine learning classification on the extracted features. As the patient performs a test movement, he/she is monitored by one or more sensors. After the test movement is finished, the sensor data is retrieved by the main program, which performs some pre-processing on the sensor data to make it more manageable for further processing. The next stage is to extract features from the data, which must represent meaningful information about the test movement (such as joint angles, *etc.*). Once these features are extracted, they can be used as inputs to a machine learning algorithm, which classifies the movement as one of a discrete number of classes. The classification is then used to define the score of the test movement. These stages are described in further detail in the following subsections.

### 2.1. Sensors

**Figure 3:**
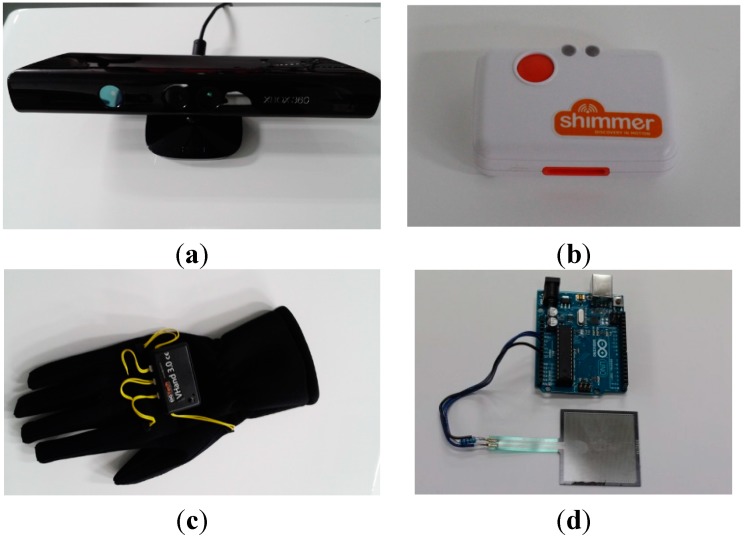
The main sensors we used to record movement with our system. The Kinect in (**a**) and the inertial measurement unit in (**b**) can be used for motion capture, the glove in (**c**) can monitor the state of the hand and fingers, and the pressure sensor in (**d**) can be used to measure grip strength.

The first stage of the framework is to obtain information about patients performing test movements through sensors. This involves deciding which sensors to use. Since all conventional upper-limb assessments are mainly based on gesture recognition by a clinician, we opted to start with the Microsoft Kinect sensor. The Kinect has proven to be one of the most promising sensors for general gesture recognition due to its relatively cheap cost and its ability to detect the limb movements of up to two people simultaneously with reliable accuracy. Multiple studies have shown that the Kinect can be used to distinguish between different human poses successfully [10,11], to monitor gait [12] and to detect specific actions, such as falling or performing aggressive actions [13,14]. Moreover, several studies have implemented systems that utilized the Kinect for stroke patient rehabilitation [15,16,17,18]. The Kinect, shown in Figure 3a, can accurately track joint positions of a patient in 3D space, which makes it very useful for various upper-limb assessments. It can be used with the Kinect SDK, which provides functionality to access skeleton data (joint position data) from the depth sensor, the video feed, and the microphone (which includes speech recognition capability). The Kinect offers a convenient method for identifying features specified in standard evaluations, such as joint angles.

One core idea of the proposed framework is to combine Kinect data with sensor data from other sensors for better automated upper-limb assessment. The idea comes from the following disadvantages with the Kinect:

(1) It is unable to detect more subtle movements such as twisting motions (supination and pronation), and shakiness, which are essential for upper-limb assessment.
(2) Its readings can be noisy and inaccurate when there is any level of occlusion. Moreover, the readings are problematic for patients bound to a wheelchair. Since the Kinect uses infrared readings, it does not have the ability to measure many details that can be observed through a video feed. From the Kinect’s perspective, an armrest of the wheelchair can look very similar to an arm, so it has difficulty distinguishing a patient’s arm from the armrest.
(3) It can be inaccurate at tracking hand position, and does not currently support finger tracking. Hand function is important for upper-limb motor function.

In order to overcome the limitations above, we opted to use a Shimmer inertial measurement unit (IMU, Shimmer, Dublin, Ireland) as shown in Figure 3b and/or a glove sensor (DG5-VHand glove 3.0, DGTech, Bazzano, Italy) as shown in Figure 3c. These sensors have been used for gesture recognition with success [19,20,21,22]. Moreover, IMUs have been used with the Kinect to track upper-limb movement accurately [23,24]. The IMU, which contains 3D accelerometer and 3D gyroscope, can get movement information, such as linear acceleration, linear speed (by integrating linear acceleration), angular speed, and length of a movement (by checking speed calculated). By strapping the IMU to a patient’s wrists or to the back of their hands, we found IMUs useful for detecting supination, pronation, wrist circumduction, and shakiness by using the movement information obtained. The glove sensor contains flexion sensors sewn along the fingers. The flexion sensors allow the glove to detect how far each finger is bent. This information is useful for detecting finger flexion, finger extension, and grasping movements.

The final sensor that we investigated for use is a pressure sensor (FSR 400 series, Interlink Electronics, Westlake Village, CA, USA) shown in Figure 3d, which we used to measure patient grip strength. While the sensors that we outlined in this section can cover a wide variety of movements in many clinical instruments for the assessment, more sensors can be added to further extend coverage.

Each sensor that interfaces with the system is driven by a separate program that provides a layer of abstraction. The sensor interfaces contain the routines for collecting data from the sensor and formatting it. These interfaces then connect to the main system via a socket. This allows the main system to simply send a signal to each sensor interface to start recording or to stop recording and request the sensor data. These sensor interfaces follow a simple template that sets up the socket interface with the main system. This modularity provides the benefit of making it easy for a designer to add additional sensors.

### 2.2. Data-Preprocessing

As the sensors record the patient’s movements in the previous stage, several factors introduce noise into the sensor data. Occlusion, which is when something blocks the view of whatever the camera is trying to observe, causes a significant amount of noise in the Kinect, while inadvertent shaking in the patient’s arms may cause IMU data to appear noisy. Another issue with the sensor data is the amount of variability in patient movements, such as speed of the movement. Hence, pre-processing on the sensor data is necessary before we can extract useful information from it. We have implemented pre-processing routines that we have found to be commonly needed for Kinect data and IMU data.

Our primary focus for the Kinect data was to remove the speed of the movement as a factor that may affect the scoring. In all clinical instruments for upper-limb assessment, the speed is typically not a measure to evaluate patient’s motor function. Since people perform movements at different speeds, we increased or decreased the speed of the movement so that movements from different people would appear to happen at the same speed. This was accomplished by scanning the set of Kinect skeleton frames and calculating the distance that the wrist had traveled between each frame. We used the wrist as the point of reference for movement speed because the wrist is the furthest extremity of the upper body that we could measure without too much noise; the wrist moved the furthest distance compared to the other joints. For adjacent frames where there was not a significant amount of movement, the routine deletes a number of the frames. After this step, when the frames are played back, the skeleton frames appear to represent a sped-up movement with relatively uniform speed throughout the movement. The next step is to set the total number of frames to a constant number (a specified value for each test movement). This is done by determining the number of frames that need to be added or removed. Then, frames are either removed or repeated at even intervals.

Other Kinect routines include a routine for representing a limb as a normalized 3D vector and a routine for calculating the angle of a joint. These routines are discussed in further detail in Section 2.3. We also implemented a routine to analyze a set of skeleton frames and identify the frame in which the wrist is at the highest or lowest point. This can be useful for scoring many movements such as shoulder or elbow flexion, where we want the system to observe how high the patient can raise their hand while flexing the appropriate muscles.

For the IMU, one of the most frequent routines we used was median filtering. Median filtering [25] is a simple approach that smooths the accelerometer and gyroscope readings to enable further pre-processing to be performed. After this, we created a routine to identify the range of twisting motions. This routine could be used to calculate the range of supination, pronation, and wrist circumduction.

Other sensors can reuse many of the pre-processing routines that we have implemented, but they will often require additional pre-processing routines to allow for feature extraction. Our gloves and pressure sensor used the same median filtering routine as the IMU, but included extra test-specific pre-processing such as calculating average sensor values.

### 2.3. Feature Extraction

The pre-processing phase of the proposed framework opens up the ability to more easily extract relevant features from the sensor data. Features should represent meaningful information that can be used to differentiate between the different classes of data. For instance, if we design a system that determines a person’s pose, we may attempt to extract features such as the positions of the arms and legs relative to the rest of the body, since this may be enough information to determine one of a few different possible poses. This feature data is used as input to the classification algorithm, which decides the final movement score based on the feature values.

Feature extraction routines are unique for each test movement. This is because different movements have different requirements for obtaining each possible score. For example, testing for the ability to flex the biceps requires us to look at a subject’s elbow joint angle, while testing for the ability to supinate the wrist require us to focus on the subject’s wrist. Despite this, most routines for the test movements re-use a common set of features. An example of this would be elbow flexion and elbow extension, which both look at the elbow joint angle. We have created a set of feature extraction routines that each provide information about an upper body feature. To add support for additional tests, an additional feature extraction routine must be created for the system to handle that specific case. This can generally be done by using a combination of the feature extraction routines that we present in the following paragraphs.

**Figure 4:**
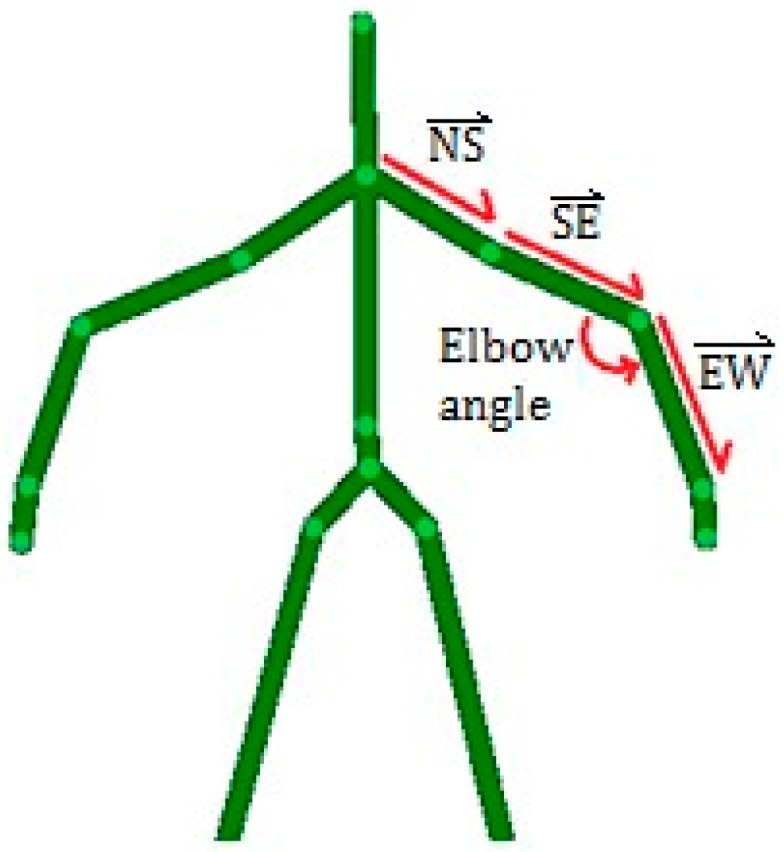
A depiction of the skeleton data gathered by the Kinect with the features we extracted from that data. The limbs between the neck (N), the shoulder (S), the elbow (E), and wrist (W) are converted to 3D unit vectors. The elbow angle is calculated based on the vectors of the forearm and upper arm.

From Kinect data, the main features that may present interest for automating test cases are limb orientations and joint angles. Limb orientations are calculated by representing the two adjacent joints as a 3D vector. This vector is normalized to remove the slight amount of variation that may occur from noise or differing heights among patients. Joint angles are calculated by using the dot product of the 3D vectors of the adjacent limbs, as follows:

(1) $$\theta =\arccos\left(S^{T}\cdot I\right)=\arccos\left[\left(s_{1}i_{1}\right)+\left(s_{2}i_{2}\right)+\left(s_{3}i_{3}\right)\right]$$

where $S=\left[s_{1}\;s_{2}\;s_{3}\right]$ and $I=\left[i_{1}\;i_{2}\;i_{3}\right]$ denote the 3D vectors of the superior limb and the inferior limb, respectively. The typical features extracted from Kinect data are illustrated in Figure 4. To calculate the elbow angle shown in Figure 4, the upper arm SE would be the superior joint S while the forearm EW would be the inferior joint I.

**Figure 5:**
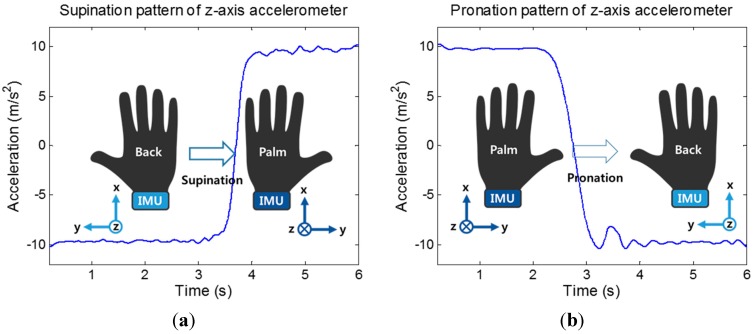
Z-axis accelerometer readings when subject’s elbow supination/pronation movement occur. (**a**) Supination (**b**) Pronation

For the IMU, the most common type of movement for which we gathered features were supination and pronation. This is because there are multiple tests that require these types of movements in different poses. To measure this type of movement, the IMU is attached to the wrist, positioned similarly to a wristwatch. As the supination or pronation occurs, the Z accelerometer readings appear as shown in Figure 5, mainly due to gravity. The range of supination or pronation was determined by smoothing the sensor readings with median filtering and using the difference of the maximum and minimum sensor value from the movement. This routine is also useful for detecting the range of wrist circumduction and dorsiflexion. Another routine that we implemented was to check the shakiness of a movement by comparing the IMU data with the smoothed (via median filtering) version of itself. Specifically, we gathered accelerometer readings of such movements, which appeared to be much less smooth for shaky movements than for smoother movements. The overall sum of the differences between these data sets results in a higher value for more shaky movements and a lower value for smoother movements. The third routine we implemented was to determine the amount of movement that occurred for a test movement, measured at the wrist. This is necessary for test movements where there is slight occlusion from the Kinect. To do this, the routine gathers gyroscope readings from the movement and establishes the resting value, or the value of the readings when there is no movement. The routine then integrates over the readings that appear above this line, resulting in a higher value for gyroscope readings with more movement.

The glove and pressure sensors required very basic feature extraction routines. For the glove sensor, our routines find the point where the fingers are most flexed or extended (depending on the test being performed). The features are simply the sensor values that appear at this point. For the pressure sensor, the feature extracted is the maximum value that appears in the list after smoothing.

In summary, the feature extraction routines that we created are briefly outlined in Table 1. As stated previously, the routines that gather extracted features for each test movement combine data from the feature extraction routines outlined in this section. The features from these feature extraction routines are added to a list that is then used as the input to the machine learning algorithm. For example, if we wanted to measure a joint angle and two limb orientations for a particular frame as the feature set for a test movement, it would have seven dimensions: One joint angle and two sets of 3-dimensional vectors representing each limb orientation. It is worth noting that all pre-processing and feature extraction routines mentioned in this paper have been fully automated and designed to run in real-time. The run time for the entire process of pre-processing sensor data, extracting features, and classification took between 0.7 and 1.5 s.

**Table 1:** An outline of the feature extraction routines implemented to analyze upper limb movement.

Feature	Dimensions	Feature Values
Limb orientation	3	X,Y,Z unit vector indicating the direction the limb is pointing
Joint Angle	1	Angle of the joint in radians
Supination and Pronation	1	Difference between max and min values of the Z accelerometer
Movement smoothness	1	Difference between movement accelerometer readings and the smoothed readings
Amount of movement	1	Integral of gyroscope readings for the movement
Grip strength	1	Maximum sensor value after smoothing
Finger Flexion and Extension	5	Maximum sensor values for each flexion sensor after smoothing

### 2.4. Data Classification

The features extracted from the sensor data can be used as input into a classification algorithm. One of the most popular machine learning algorithms used for gesture recognition is the Support Vector Machine (SVM) [26], which is a statistical machine learning algorithm that can be used to classify linear data. SVM also supports nonlinear data classification by using a kernel function to map nonlinear data into a higher dimensional feature space, which can make it possible to perform linear separation.

Another popular machine learning algorithm for this type of application is the artificial neural network, or a specific type called a Backpropagation Neural Network (BNN) [27]. They consist of a network of interconnected neurons that are divided into three layers: The input layer, hidden layer, and output layer. There is a weighted connection between each node and every node on an adjacent layer. To obtain the result of a classification, feature data is placed into the input layer and “fed forward” to get an output from the output layer. The primary challenge of using artificial neural networks is to adjust the weights and biases through training to produce meaningful output for any input. A common approach to do this is Backpropagation, an iterative algorithm that uses the gradient descent algorithm on the neural network’s error function to adjust the weights and biases.

We opted to investigate machine learning algorithms because despite the training and testing process, the algorithms can be reused for multiple tests without going through heavy re-implementation. The SVM and BNN machine learning algorithms have been demonstrated to work with similar sensor data in many previous works [11]. We chose to demonstrate these two algorithms to see if there was a significant difference in accuracy with classifiers that used different approaches (statistical classification *vs.* neural network). The only test-specific changes to make are the features to investigate, which affects the number of input and hidden layer nodes in the BNNs. This is an advantage over approaches that may require a different algorithm to be created for every single test movement. For example, the authors in [9] implemented a different approach to the automation of stroke assessment by using PCA to construct joint angle profiles. Machine learning provides a much more generalized approach that broadens the features from sensor data that can be incorporated into an assessment and extended without having a significant impact on complexity. Another advantage of using such classification algorithms is that the standard evaluations we are aiming to automate apply scores from a discrete set of possible values (such as 0, 1, and 2 for the FMA). Each of these possible values can easily translate to a class label of any classification algorithm.

## 3. Feasibility Evaluation

### 3.1. FMA Implementation

To demonstrate the feasibility of using the proposed framework to create a working automated assessment system, we implemented a system that focuses on automating the FMA. The FMA consists of 33 upper-limb motor tests, where the clinician asks a patient to perform a movement. As the patient performs each movement the clinician scores the movement according to the following guidelines:

- 0: the patient cannot perform the movement at all;
- 1: the patient can perform the movement partially;
- 2: the patient can perform the movement faultlessly.

As the patient performs each test movement, the scores are added up to give an overall score that represent the patient’s level of limb motor function impairment.

**Figure 6:**
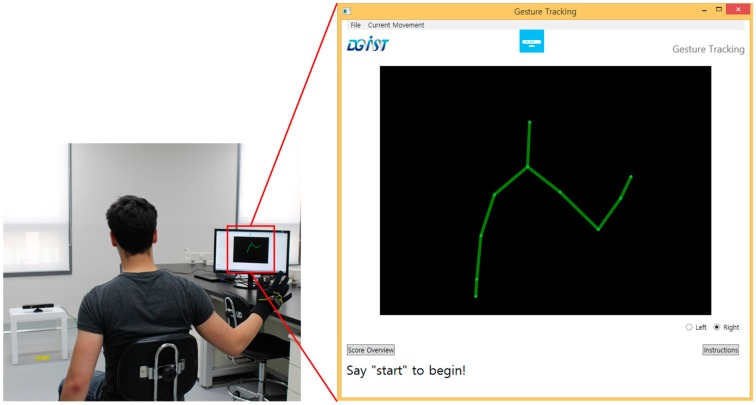
The primary user interface for our system, which shows a graphical representation of the Kinect’s sensor data.

Based on the proposed framework, the system we developed works with the Kinect, the glove sensor (which includes an IMU), and a pressure sensor. The Kinect records data at 30 Hz and connects to a computer via USB. It is positioned approximately 1.5 m in front of the subject. The glove sensor which was worn by the subject’s hand collects data through USB at 100 Hz. The pressure sensor that was placed on a table within reach of the subject measures the subject’s grip strength by using Arduino board at 30 Hz. Our implementation of the automated FMA, as displayed in Figure 6, follows the standard outlined in [28]. Based on the user interface (Figure 6), the user selects which test movement to perform, and is presented with video instructions for performing them. Recording is then started by a voice command and stops when the application automatically detects that the user has finished moving. The sensor data is then classified through SVM to obtain a final score, which is displayed by the application (Figure 6).

**Figure 7:**
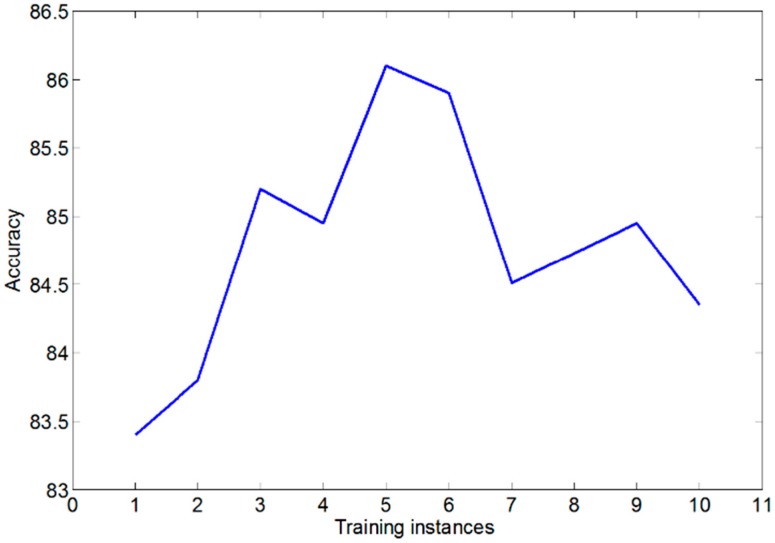
The accuracy of our classifiers given the number of training examples.

Our system can automate 24 out of the 33 (about 73%) upper-limb tests of the FMA, as summarized in Table 2. The tests that we did not support include movements that could not be measured with the sensors outlined in this paper, or were tests that require reflex activity, because those tests require some form of actuation. The feature extraction routines for these tests are all a combination of one or more of the routines mentioned in Section 2.3. Due to the different requirements for each test to be measured, each test requires a different SVM with its own training instances. The training and testing data was not scaled because we found that in our case, this had no impact on accuracy. Each of these SVMs performed best with a linear kernel. In general, when determining the number of SVM training instances, we found that the system presented the highest accuracy when we had five training instances for each possible score for each test. Note that the accuracy is defined as percentage of test movements that were classified accurately. When we used more than five training instances, the accuracy slightly decreased, which is likely a result of overfitting [29]. Overfitting is when an SVM is unable to accurately distinguish between test instances due to noise. This can happen if the model is complex and contains a lot of features, as is the case in our implementation. Figure 7 illustrates the effect of the number of training examples on the overall accuracy of our system.

**Table 2:** The FMA tests supported by our implementation of an automated assessment system.

Category	Movement
Shoulder	Abduction
	Ext. Rotation
	Abduction 0–90°
	Flexion 0–90°
	Flexion 90–180°
Elbow	Flexion
	Extension
	Pro/Supination at 0°
	Pro/Supination at 90°
Forearm	Supination
	Pronation
Hand	Finger mass flexion
	Finger mass Extension
	Grasp a
	Grasp b
	Grasp c
	Grasp d
	Grasp e
Wrist	Flex/Extension with elbow at 90°
	Flex/Extension with elbow at 0°
	Circumduction
Coordination/Speed	Tremor
	Speed

### 3.2. Protocol

In order to evaluate the basic accuracy of our implementation, an experiment with healthy subjects were conducted. Eight healthy volunteers (seven men, one woman) ranging in age from 24 to 30 years participated in the experiment. They were seated in front of the Kinect in a chair without armrests while wearing the glove sensor. We asked them to perform each test movement three times: once where they didn’t perform the movement at all, once where they performed the movement partially, and once where they performed the movement faultlessly. This was to simulate different levels of motor function (score 0, 1, and 2 of FMA) for performing the test movements.

To test accuracy, we ran this data through our classifier using training data generated by one healthy volunteer. For this test, we used five training examples for each class of each test (15 training examples per test). Since we used a publicly available SVM library, LIBSVM [30], the SVM classifiers were trained with default settings from LIBSVM, with the exception of using a linear kernel function instead of the default polynomial kernel function. We then ran tests with the same kernel function where we diversified the training data with v-fold cross validation [31]. This was done by using data from one volunteer as a testing instance while using the data from the rest of the volunteers as training instances. This was repeated for each volunteer. We ran the same tests with our BNN classifiers using both v-fold cross validation and accuracy testing with five training instances.

To further evaluate the feasibility of our implementation, we conducted a comparison experiment with actual stroke patients. Two post stroke patients (two men, 45 and 62 years old) who do not have cognitive impairment (mini-mental state examination > 24) participated in the experiment. They gave signed informed consent approved by the DGIST IRB prior to the experiment. After installing/attaching sensors, the patients were asked to perform the FMA by using the developed system; for each test of FMA, video instructions were provided in advance, and they did their best to follow the instructions. The system recorded the patients’ data in the presence of a well-experienced clinician (occupational therapist) who also scored the test movements herself. Using data from healthy subjects as training data for the classifier (obtained from the experiment with healthy subjects above), the system rated each movement of the FMA test. To evaluate accuracy, the scores from the automated assessment system were compared with the scores obtained by the clinician. After finishing all supported automated FMA tests (25 tests summarized in Table 2), the remainder (eight tests) were performed by the clinician without the system, and the time duration spent for the remainder was measured to evaluate how much time the system would save for the clinician.

## 4. Results

In the previously mentioned experiment, we tested two machine learning algorithms on the data from healthy volunteers: SVM and BNN. The LIBSVM for the SVM classification uses the “one-vs-all” approach for multi-label classification [30]. For BNN classification, we implemented a simple 3-layer neural network. As with SVM, we set up a separate BNN for each test movement. The number of input nodes was equal to the number of features and all networks had three output nodes. The scores were determined by which output node had the highest value. The number of hidden nodes was generally set to a number about halfway between the number of input nodes and output nodes.

The experimental results with the SVM and BNN classifiers, which are represented as the percentage of test movements that were classified accurately, are summarized in Table 3. The accuracy in all cases reaches at least 75%. The results show that the proposed framework can feasibly be used to perform an automated assessment. It is noteworthy that the BNN classifier has better overall classification accuracy (about 93.1%) than the SVM classifier (about 86.1%). However, the difference between two is not statistically significant (*p* > 0.05).

Of two classifiers used above, we opted to use the SVM classifier for the latter experiment with stroke patients because of its ease of implementation and extendibility given the open source implementations of LIBSVM. The comparison results between the automated and the in-person assessments are summarized in Table 4. Compared with the results from the healthy subjects, the accuracy of the automated assessment slightly deteriorates (about 24%) with both patients. To further evaluate the clinical effect of degraded accuracy, we compared the final scores of the patient to the scores assigned by the clinician, which can be seen in Table 5. Despite the lower accuracy in each FMA test, the total scores of FMA obtained by the two assessments are quite similar (over 90% agreement).

After the supported automated assessment, the clinician conducted the assessment for the eight unsupported FMA tests, and it took 5 min on average. This result shows that the proposed framework would reduce the amount of time it takes to perform a patient assessment by about 82%.

**Table 3:** Result of scoring (classification) from healthy volunteers after they performed each test.

Subject	SVM		BNN	
	Accuracy	V-Fold Accuracy	Accuracy	V-Fold Accuracy
1	93.65%	90.48%	95.24%	96.83%
2	75.76%	86.36%	87.88%	93.94%
3	84.85%	89.39%	93.06%	95.83%
4	78.79%	84.85%	81.82%	86.36%
5	89.23%	90.77%	83.10%	88.73%
6	87.88%	87.88%	93.10%	93.10%
7	95.65%	89.39%	96.00%	93.33%
8	84.13%	96.83%	90.48%	96.83%

**Table 4:** Results of scoring (classification) from stroke patients as compared to scores assigned by a clinician.

Person	SVM Accuracy
P1	54.55%
P2	68.18%

**Table 5:** A comparison of the FMA scores assigned by our implemented system and the score assigned by the clinician.

Person	SVM Score	Clinician Score	Score Accuracy
P1	29	30	96.67%
P2	30	33	90.91%

## 5. Discussion

The feasibility of the proposed framework was evaluated by implementing a system to automate FMA. The automated FMA was quite accurate with healthy subjects, but less accurate with post stroke patients. This is mainly because the training data used by the classifier in the system was collected from healthy subjects’ movements, not movements from stroke patients. The most promising method of increasing classification accuracy would be to collect movement data from stroke patients to use as training data. Another factor that impacted the final accuracy was that the clinician’s scores were used as ground truth. Since the FMA has been proven to have high inter-rater reliability [28,32], we only recruited one occupational therapist who is well-experienced in performing the FMA. However, more clinicians may allow for more rigorous accuracy analysis.

The proposed framework was designed to be used to create systems that will be deployed in relatively controlled settings in hospitals. Patients can schedule appointments to perform automated assessments with these systems. This would free up a lot of time for clinicians, since they would no longer have to perform the assessments themselves. The only step that would require the presence of a clinician would be to put the glove sensor onto the patient’s hand, which sometimes presents some difficulty due to lack of motor control in most patients’ hands. In our experience, this typically takes a negligible one or two minutes.

The sensors that we have investigated for the proposed framework are relatively cheap, which opens up the possibility for the framework to be deployed in-home environments [12,13]. Note that reliable person identification performance [33] would be required for the home environments with multiple people. This in-home assessment can further save time for clinicians as well as making things more convenient for patients. It also enables more frequent assessment that will allow clinicians to closely monitor patients remotely.

In the FMA case, we automated 25 out of the 33 upper-limb tests based on the proposed framework. The eight unsupported tests can be classified into two groups: external actuation group (biceps/triceps muscle reflex, normal reflex activity, and wrist stability at elbow 0°/90°) and fine motion group (shoulder retraction/elevation, dysmetria). The former group requires some kind of external actuation that cannot be provided by a sensor-based framework. A robotic device may be the solution, but it would significantly increase the cost and potentially reduce the time effectiveness of the automated assessment. The latter group is only feasible by adding several additional high-performance (potentially high-cost) sensors for measuring fine motions, and the use of such sensors also results in additional setup time. To maximize the cost/time-effectiveness with large coverage, we decided to cover 25 tests of FMA through the framework.

The design of the proposed framework also opens up the possibility for automating tests from other standard evaluation methods, such as the ARAT, WMFT, and NIHSS. Adding a new automated test to an existing system is a matter of creating a single feature extraction routine and generating training data. In addition to the type of automated tests, the framework allows for the customization to choose a machine learning classification algorithm. Our tests indicate statistically insignificant differences in accuracy with SVM and BNN, but other algorithms may also be applied and tested. This customizability allows the developer to create custom test sequences to monitor the conditions of their patients.

This paper is limited in that it used a small population of post stroke patients, thus training data from healthy volunteers was used to implement the automated FMA. Since this study was designed to show the feasibility of the proposed framework for the automated upper-limb assessment, we have started to collect training data from more stroke patients with a clinical partner. This planned study is meant to compare the in-person FMA and the automated FMA with a broader range of participants, including patients with more variable levels of upper limb impairment. Additional future plans include providing multi-modal instructions for subjects with cognitive impairment and considering safety issues to extend the proposed idea to an in-home assessment.

## 6. Conclusions

This paper proposed a novel framework for automating the assessment of upper-limb motor impairment. The main contribution of this work is to provide a framework that can be used to create a series of tests that can evaluate the upper-limb function of a patient without clinician supervision. Using this framework, we implemented a system that automates 73% of the upper limb portion of the FMA. The system implemented with our approach has an acceptable accuracy and can be used to save approximately 25 min per patient for clinicians. This framework allows stroke patients to be evaluated more frequently at lower cost.
